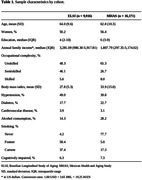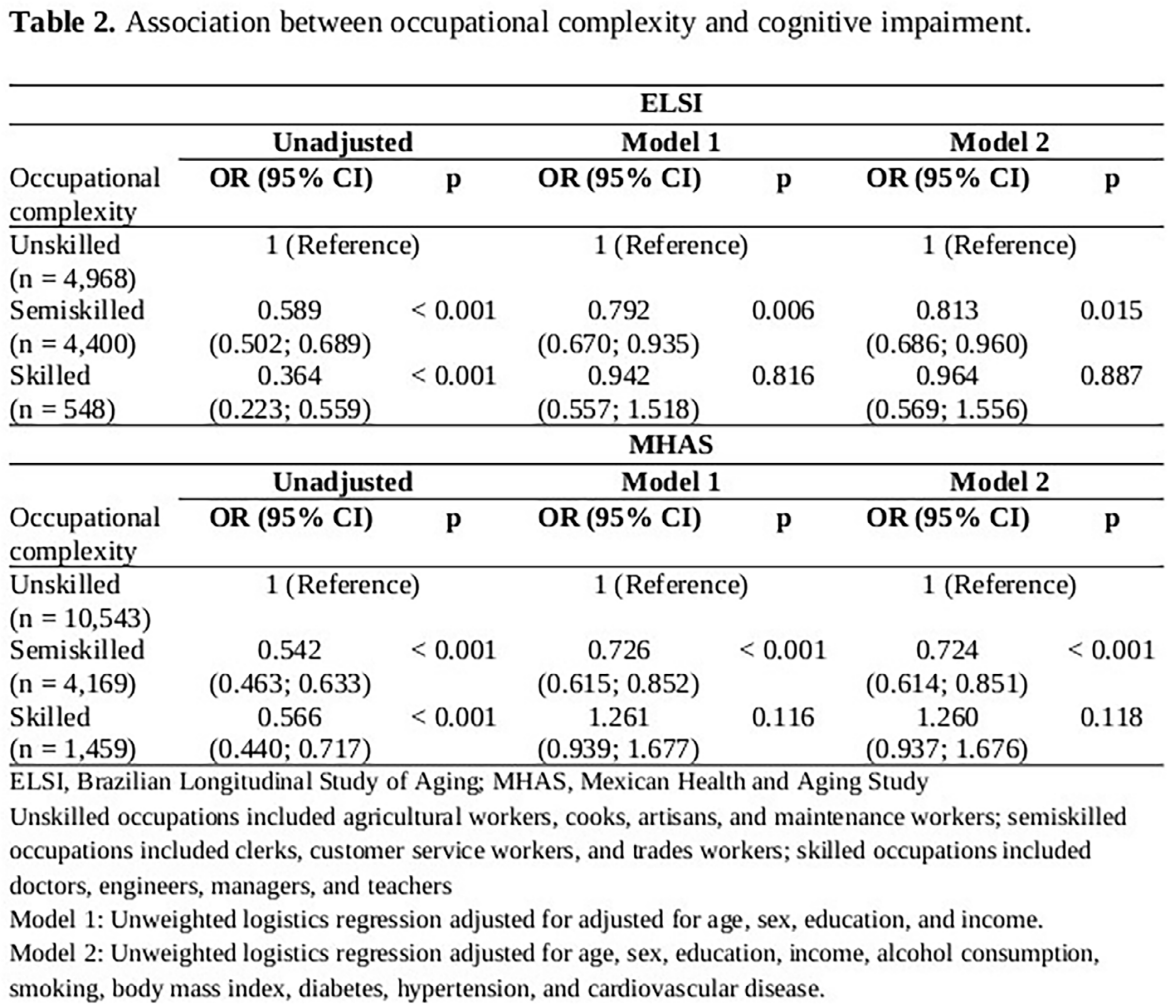# Main occupation complexity and later‐life cognitive function among older adults in Brazil and Mexico

**DOI:** 10.1002/alz.088157

**Published:** 2025-01-09

**Authors:** Natalia G Gonçalves, Gabriela Mininel de Medeiros, Jaqueline Contrera Avila, Laiss Bertola, Cleusa P Ferri, Rebeca Wong, Claudia Kimie Suemoto

**Affiliations:** ^1^ University of São Paulo Medical School, Sao Paulo Brazil; ^2^ University of São Paulo, São Paulo, SP Brazil; ^3^ University of Massachusetts Boston, Boston, MA USA; ^4^ Universidade Federal de São Paulo (UNIFESP), São Paulo, São Paulo Brazil; ^5^ Universidade Federal de São Paulo (UNIFESP), São Paulo, São Paulo/SP Brazil; ^6^ University of Texas Medical Branch, Galveston, TX USA; ^7^ University of São Paulo Medical School, São Paulo, São Paulo Brazil

## Abstract

**Background:**

Occupation complexity during adulthood may contribute to cognitive reserve in later life. Research on occupational complexity and cognitive function has focused on high‐income countries, where there is a large proportion of individuals with complex occupations. Thus, it is important to investigate this association in the context of low‐ and middle‐income countries where there is greater variance in occupation complexity and where there is a higher proportion of lower‐complexity occupations due to educational and other socioeconomic limitations. We use nationally representative data from the two largest countries in Latin America, Mexico and Brazil, to assess the association between main occupation complexity and cognitive function.

**Method:**

The sample included adults 50 years or older from the 2018 waves of the Brazilian Longitudinal Study of Aging (ELSI) (n = 9,916) and the Mexican Health and Aging Study (MHAS) (n = 16,171). Participants were classified as cognitively impaired or not impaired using cognitive performance scores and a regression‐based approach compared to a normative sample. Main occupation was categorized as unskilled (agricultural workers, cooks, artisans, and maintenance workers), semiskilled (clerks, customer service workers, and trades workers), and skilled (doctors, engineers, managers, and teachers). We used logistic regression models to estimate the association between occupational complexity and cognitive function adjusted for sociodemographic variables, chronic diseases, and health behaviors.

**Result:**

Participants of the ELSI had a mean age of 64.0±9.6 years, median years of education of 4 years, 50% were women, 48% had unskilled occupations, and 6% were categorized as cognitively impaired (Table 1). Participants of the MHAS had a mean age of 62.8±10.3 years, median years of education of 6 years, 56% were women, 65% had unskilled occupations, and 7% were categorized as cognitively impaired (Table 1). Participants with semiskilled occupations had lower odds of cognitive impairment than those with unskilled occupations in both cohorts (Table 2). There was no association between main lifetime skilled occupations and cognitive impairment in either cohort (Table 2).

**Conclusion:**

Higher occupational complexity may contribute to later‐life cognitive reserve. The results of this study suggest that increasing opportunities for higher‐complexity occupations in Latin America may benefit efforts to prevent cognitive impairment in this region.